# The Emotion Regulation Flexibility Questionnaire: A Rasch-Validated Version for Children

**DOI:** 10.3390/jintelligence14070129

**Published:** 2026-07-01

**Authors:** Roberto Burro, Giada Vicentini, Giada Mignolli, Anna Maria Meneghini, Daniela Raccanello

**Affiliations:** Department of Human Sciences, University of Verona, 37129 Verona, Italy; roberto.burro@univr.it (R.B.); giada.vicentini@univr.it (G.V.); giada.mignolli@univr.it (G.M.); anna.meneghini@univr.it (A.M.M.)

**Keywords:** emotion regulation flexibility, malleability of emotions, well-being, children, Rasch analysis

## Abstract

The ability to effectively use different emotion regulation (ER) strategies across situations—i.e., ER flexibility (ERF)—plays a key role in emotional intelligence and social functioning. However, it has rarely been studied in young people, partly due to the lack of age-appropriate measurement instruments. This study investigated children’s perceptions of ERF by adapting an adult scale and proposing the 10-item Emotion Regulation Flexibility Questionnaire for Children (ERFQ-C). A sample of 346 children aged 9–12 years was recruited, who completed the ERFQ-C along with questionnaires assessing emotion malleability and school-related well-being. First, the analyses confirmed the scale’s unidimensional structure and measurement invariance across sex and school level. Second, the scale showed adequate fit to the Rasch model, and item scores were converted into Rasch scores. Third, path analyses indicated that emotion malleability was positively associated with ERF perception, which, in turn, was positively associated with school-related well-being. Notwithstanding the limitations of self-report data, the instrument can be used in future research to advance theoretical understanding of the intertwinement between perceived ERF and other constructs related to children’s functioning (e.g., emotional intelligence) and inform interventions aimed at improving youth well-being.

## 1. Introduction

Emotion regulation flexibility (ERF) refers to the capacity to modulate and deploy regulatory strategies in ways that are appropriately calibrated to situational and contextual demands (Aldao et al., 2015). ERF is inherently linked to emotion regulation (ER; Gross, 1998, 2015), a core component of emotional intelligence as conceptualized in the ability-based framework proposed by Salovey and Mayer (1990) and Mayer and Salovey (1997)—that is, the capacity to perceive, use, understand, and manage emotions (Mayer et al., 2016). Consistent with this view, a growing body of empirical research has documented systematic associations between ER processes and emotional intelligence (Peña-Sarrionandia et al., 2015), and emerging evidence suggests that higher emotional intelligence is also associated with greater ERF in daily life (Double et al., 2022). Although scholarly attention to ERF has grown substantially in adult populations over recent years, considerably less research has focused on children (Haag et al., 2024), partly because of the lack of age-appropriate assessment instruments.

To address this gap, an adult 10-item self-report measure, the Emotion Regulation Flexibility Questionnaire (ERFQ; Monsoon et al., 2022; Italian version, IT-ERFQ, by Mignolli et al., 2025), was adapted to develop the Emotion Regulation Flexibility Questionnaire for Children (ERFQ-C). To further evaluate the scale’s psychometric properties and ensure adherence to the principles of fundamental measurement, Rasch analysis (RA; Andrich, 1988; Bond & Fox, 2007) was conducted. Finally, to extend current theoretical understanding of ERF perception in younger populations, its associations with two emotion-related constructs were examined: beliefs about the malleability of emotions and school-related well-being. Overall, the present study contributes to the literature by addressing three primary gaps: (a) the lack of instruments specifically designed to assess perceived ERF in children; (b) the scarcity of perceived ERF measures developed and validated according to fundamental measurement principles; and (c) the limited empirical evidence concerning perceived ERF’s correlates in youth.

### 1.1. Emotion Regulation and Emotion Regulation Flexibility

Gross (1998, 2015) conceptualized ER as the process through which individuals influence which emotions they experience, when these emotions arise, and how they are experienced and expressed. Central to Gross’s definition is the assumption that emotions are multicomponent processes unfolding over time; accordingly, regulation involves modifications in the temporal dynamics of emotional responding. In the context of ER, scholarly literature has traditionally distinguished between adaptive strategies (e.g., reappraisal) and maladaptive strategies (e.g., expressive suppression; Gross, 2015). Building on this premise, Gross (1998, 2015) introduced the process model of ER (PMER), which differentiates regulatory strategies according to the stage of the emotion-generative sequence at which they exert their primary effects. Within this framework, emotions do not inherently require regulation in every circumstance; rather, regulatory efforts become necessary when emotional responses interfere with the pursuit or enactment of goal-directed behavior (Gross, 1998, 2015). In any case, ER is essential for individuals’ mental health and proper social functioning (Gross, 1998, 2015). Moreover, when ER functions effectively, an individual’s resources—particularly during childhood—can be channeled into growth and development (Daniel et al., 2020).

Grounded in this conceptualization, Aldao et al. (2015) defined ERF as “the degree of covariation between ER variability and changes in the environment, where the environment might consist of external events and/or appraisals of emotional reactions to such events” (p. 268). From this perspective, ERF is considered adaptive insofar as its deployment increases individuals’ capacity to pursue and attain personally meaningful goals (Aldao et al., 2015). Therefore, focusing on ERF redirects attention from the primacy of any single regulatory strategy and instead emphasizes the context-sensitive and flexible use of multiple strategies.

Bonanno and Burton (2013) identified three theoretically distinct elements that are involved in the implementation of ERF: context sensitivity, repertoire, and feedback responsiveness (Kalokerinos & Koval, 2024). First, context sensitivity entails the ability to evaluate contextual characteristics and situational demands in circumstances that may elicit emotions requiring regulation. Second, repertoire involves the availability of a wide range of regulatory strategies on which the individual can rely (De France & Hollenstein, 2017). Third, feedback responsiveness involves the ability to monitor the effectiveness of a selected strategy and adjust or replace it when necessary.

Thus, within this framework (Bonanno & Burton, 2013), having a wide ER repertoire available is not equivalent to being flexible. Indeed, as suggested by Aldao et al. (2015), flexibility implies the ability to vary strategies, calibrating them to contextual changes. This view is also consistent with Gross’s (1998, 2015) process model, in which regulatory strategies are embedded within temporally unfolding emotion-generative processes. Therefore, in the present study, ERF is conceptualized not as the mere number of strategies available to the child, but as children’s perceived capacity to use different strategies in a context-sensitive and feedback-responsive manner.

From a developmental perspective, ER undergoes substantial changes across childhood and adolescence. Development is characterized by the progressive acquisition and refinement of ER strategies (Zimmer-Gembeck & Skinner, 2011). Early in childhood, regulation relies predominantly on externally supported and behavioral strategies (e.g., seeking support, physically withdrawing from situations). With age, children’s repertoire gradually expands to include more autonomous, intrapersonal strategies and cognitively mediated forms of regulation (e.g., reappraisal or attentional disengagement; Garnefski et al., 2001; Phillips & Power, 2007; Zimmer-Gembeck & Skinner, 2011). Thus, developmental change is associated with an increasing diversity of available strategies, providing a broader repertoire from which individuals can potentially draw. However, the expansion of the repertoire does not, in itself, imply greater flexibility, which additionally requires the ability to select, coordinate, and adapt strategies in relation to contextual demands. This distinction is particularly relevant in middle childhood, when regulatory capacities are still developing.

From the same perspective, it is also important to consider that children’s emotional functioning is strongly influenced by their subjective representations of control and value. For example, according to prominent approaches such as the control-value theory of achievement emotions (Pekrun, 2006, 2024), emotions are primarily shaped by individuals’ perceived control over situations and the value attributed to them. Recent extensions of this framework (e.g., the ER in Achievement Situations, ERAS, model; Harley et al., 2019) further highlight that ER processes operate in conjunction with these representations, with regulatory strategies influencing emotions both directly and indirectly through perceived control and appraisal processes. Within this perspective, children’s self-reported representations of their regulatory abilities may play a central role in shaping their emotional experiences and behaviors, even when actual regulatory performance is still developing.

Finally, it is worth noting that the literature recognizes both implicit and explicit forms of ER (Gross, 2015; Gyurak et al., 2011) that interact to influence individuals’ behaviors and choices. However, most studies on ER and flexibility in ER have focused on explicit processes, exploring the strategies individuals consciously rely on to modify the type, intensity, and/or duration of their emotions.

### 1.2. Correlates of Emotion Regulation Flexibility

ERF has been investigated mainly with adults. Cross-sectional studies indicated that ERF is usually associated with enhanced well-being, adjustment, and positive affect (e.g., Bonanno et al., 2004; Doorley et al., 2020; Eldesouky & English, 2021; Grommisch et al., 2020) on the one hand, and better mental health, including diminished distress and other psychopathological symptoms (e.g., Doorley et al., 2020; Goodman et al., 2021; Southward et al., 2018) on the other hand. Similar effects were confirmed with longitudinal approaches. Battaglini et al. (2023), for example, found that higher ERF was associated with increased positive affect and reduced negative affect, drawing on two-week daily diaries. Considering the processes underlying ERF, prior literature also indicated that the selection of ER strategies within a given context depends on individual differences, such as executive functioning (Toh & Yang, 2024). For example, cognitive control processes (i.e., inhibiting prepotent responses, updating information in working memory, and shifting mental sets) are considered crucial for the dynamic adaptation to varying emotional contexts, allowing individuals to effectively monitor their emotional state, maintain an adaptive strategy, or switch and stop strategies when environmental demands change (Pruessner et al., 2020). While these processes are well-documented in the adult population, much less is known about the correlates of youth’s ERF—with relatively more studies on adolescents than children beyond preschool age (Haag et al., 2024)—mainly due to inconsistencies in its conceptualization and assessment tools.

Some published works on the ERF of youth can be examined by applying Bonanno and Burton’s (2013) classification directly. On the whole, they used a variety of methodologies (Braet et al., 2026; Haag et al., 2024). A few studies examined the ability to adjust ER strategies in response to contextual cues, a capacity related to both context sensitivity and feedback responsiveness. For example, Parsafar et al. (2019) had children aged 4–11 watch a negative movie with no ER instructions or guidance to use distraction or reappraisal. Distraction was mentioned more often when children reported negative emotions, suggesting early awareness that disengagement helps manage unpleasant feelings. Using ecological momentary assessment (EMA), McKone et al. (2024) found that adolescent girls adjusted their ER strategies based on perceived control over interpersonal stressors. Higher perceived control was associated with primary control strategies (aimed at directly eradicating the stressors; e.g., problem-solving, seeking support) rather than secondary control strategies (aimed at managing the emotional response to the stressor; e.g., reframing, acceptance).

The repertoire has been studied using at least three different ways, in some cases also with young people (Braet et al., 2026). A first way is to consider the number of strategies an individual usually relies on, for example, in response to emotion-evoking situations. In one study, Quiñones-Camacho and Davis (2018) summed the strategies reported by 7-to-11-year-olds in interviews about negative episodes, and found that higher resting respiratory sinus arrhythmia, a measure of physiological regulation, was most strongly associated with lower anxiety among older children with a wider repertoire of fear-related strategies. A second way implies measuring individuals’ trait levels of ER and identifying ER profiles through person-centered analyses. With this approach, De France and Hollenstein (2019) examined strategy use throughout the life course, beginning in adolescence. While cognitive reappraisal was beneficial for relationship quality only among older adults, expressive suppression was positively associated with this variable in the adolescent and young adult cohorts, supporting the idea that the adaptiveness of ER strategies varies across developmental stages and specific social contexts. In another study, Lougheed and Hollenstein (2012) indicated that for 13-year-olds, a limited repertoire would be associated with internalizing problems such as depression, general anxiety, and social anxiety. A third way utilizes EMA to extract ER profiles of how individuals regulate emotions in everyday life. Applying this latter approach, Braet et al. (2026) identified three latent profiles of 12-year-olds’ ER strategies (distraction, cognitive reappraisal, engagement, expressive suppression, rumination, social sharing, and self-compassion). Mental health varied across groups: the no-ER group (minimal strategy use) showed the most favorable outcomes, the multi-ER group (moderate use with fewer adaptive strategies) the poorest, and the high adaptive group (frequent adaptive strategies) a mixed pattern, combining higher positive affect with elevated negative affect. According to the authors, these findings question the assumption that a broader ER repertoire is inherently advantageous, highlighting the role of repertoire composition (i.e., the specific strategies it comprises) and its alignment with contextual demands.

In sum, independent of conceptualizations and methodologies, current literature on adolescents (and sometimes younger participants) is delineating clear associations between ERF and positive outcomes across a large range of indicators, related also to clinical outcomes, such as lower depression and anxiety or fewer problem behaviors, and social outcomes, such as better relationships with caregivers and peers (Haag et al., 2024). However, notwithstanding the emerging literature on youth’s ERF, the examination of all three ERF components together (Bonanno & Burton, 2013) and the study of correlates such as malleability beliefs about emotions and well-being remain relatively unexplored in children.

### 1.3. Emotion Regulation Flexibility as a Mediator Between Malleability Beliefs and Well-Being

Malleability beliefs are central to research on implicit theories—i.e., beliefs individuals hold about whether abilities and personal traits are malleable or fixed (Blackwell et al., 2007; Dweck, 2006). Conceptually, malleability beliefs differ from ERF in that they refer to whether children perceive emotions as changeable or controllable, whereas ERF concerns the perceived ability to use, select, and adjust ER strategies across situations.

Early work in this area focused on intelligence, showing that individuals who view intelligence as fixed are less likely to engage in self-regulatory processes than those who consider it malleable and improvable (Burnette et al., 2013). More recently, research has extended these ideas to emotions (Ford & Gross, 2019). Evidence suggests that individuals who perceive emotions as uncontrollable are less likely to use adaptive ER strategies (De Castella et al., 2013; Tamir et al., 2007; Zimmermann et al., 2021). Similarly, studies with children and adolescents show that beliefs in the malleability of emotions are positively associated with well-being, cognitive reappraisal, life satisfaction, and resilience, and negatively related to depressive symptoms, suppression, aggressive behavior, borderline personality features, and other mental health problems (Crawford et al., 2021; De France & Hollenstein, 2021; Dickerson et al., 2018; Ford et al., 2018; Jiang et al., 2022; McLachlan et al., 2022; Romero et al., 2014; Schleider & Weisz, 2016; Skymba et al., 2022). Raccanello et al. (2025), distinguishing 7-to-9-year-olds’ beliefs about positive and negative emotions, also found positive correlations between malleable beliefs and well-being, coping, and inhibition. In light of this knowledge, malleability beliefs have the potential to have a constructive impact on ER processes, including ERF.

According to the World Health Organization (1948), psychological well-being comprises a broad range of individual factors pertaining to the cognitive, emotional, and bodily domains. In line with this, school-related well-being can be defined as the psychological condition in which positive cognitions and emotions predominate over negative cognitions and emotions in the lives of members of the school context (Hascher, 2007). Some research has shown that this condition is associated with other educational outcomes for children and adolescents, for example, adaptability, achievement emotions, school performance, and behavioral conduct (Kirkcaldy et al., 2004; Putwain et al., 2020; Raccanello et al., 2025). Moreover, emotion malleability beliefs were positively associated with ER in 7-to-9-year-olds (Raccanello et al., 2022). Therefore, expanding knowledge of factors associated with school-related well-being can be a significant step toward informing evidence-based interventions to foster it.

In line with PMER assumptions (Gross, 1998, 2015), Monsoon et al. (2022) showed that adults’ ERF perception mediates the relation between malleability beliefs—in terms of emotion control, i.e., the degree to which emotions are viewed as malleable experiences that can be intentionally regulated (Tamir et al., 2007)—and distress (however, their design was cross-sectional). In particular, emotion control was associated with improvements in ERF perception, which, in turn, were negatively related to distress. This theoretical knowledge has a relevant applied impact, as beliefs about the malleability of emotions are assumed to motivate individuals to engage in ER efforts (Ford & Gross, 2019) and, moreover, can be modified (McLachlan et al., 2022; Smith et al., 2018; Specker & Nickerson, 2024). For example, providing instruction in either ER flexibility or distraction proved beneficial for anxious individuals (Specker & Nickerson, 2024). Nevertheless, to our knowledge, the mediating role of ERF perception in the relation between malleability beliefs and well-being has not been investigated among younger participants, partly because of the limited availability of instruments to assess perceived ERF in children.

### 1.4. Measurement of Emotion Regulation Flexibility

Despite broad theoretical agreement that flexible ER is a central adaptive capacity, its assessment has lacked uniformity, reflecting inconsistencies in its conceptualization (Aldao et al., 2015; Kashdan & Rottenberg, 2010). To fill this gap, Monsoon et al. (2022) developed a 10-item questionnaire used for adults (for an Italian adaptation, IT-ERFQ, see Mignolli et al., 2025). Their self-report scale was designed to capture adaptive variability in ER (Aldao et al., 2015), including the ability to match strategy use to contextual cues (e.g., “I use different types of ER strategies for different types of situations or problems”), draw on a diverse repertoire of strategies (e.g., “I use many different strategies to regulate my emotions”), and discern the appropriate timing and duration of their use, modifying them when necessary (e.g., “When an emotion regulation strategy does not work, I try to use a different one”). This item pool captures key elements of ERF as conceptualized by Bonanno and Burton’s (2013) tripartite model, encompassing, respectively, context sensitivity, repertoire, and feedback responsiveness. However, notwithstanding the relevance of investigating ERF perception with children (Haag et al., 2024), there are no versions of the ERFQ for younger individuals.

Children as young as 6 years of age can already complete self-report measures of health-related processes (Riley, 2004). Self-report instruments are well-suited to assessing ER because they capture internal states that are not directly observable (Pekrun & Bühner, 2014). They are efficient for developmental samples (Junghänel et al., 2022; Phillips & Power, 2007; Rydell et al., 2007) and can enhance ecological validity when used in daily-life designs (Aldao et al., 2015; English & Eldesouky, 2020). Nevertheless, they are vulnerable to social desirability and recall biases (Pekrun & Bühner, 2014). Overall, they remain essential for assessing the subjective dimensions of ER and can be used within multi-method approaches to triangulate findings.

More broadly, an important distinction in the ER literature concerns the difference between self-reported regulatory beliefs or perceived abilities and observed regulatory behavior. This distinction is particularly relevant in the present study, as the ERFQ-C captures children’s subjective representations of their regulatory flexibility, which may partly reflect beliefs about regulation rather than directly observed regulatory performance. Although self-report measures provide valuable access to individuals’ internal representations of their regulatory processes (Pekrun & Bühner, 2014), they may only partially correspond to how regulation is enacted in real time and across contexts. For this reason, future research should triangulate self-report data with other methodologies, such as behavioral observations or EMA, to better capture the dynamic and context-dependent nature of ER processes (Aldao et al., 2015; English & Eldesouky, 2020).

Therefore, taking into account previous literature, a version of the ERFQ for children, namely the ERFQ-C, was developed, and its psychometric rigor was then enhanced by applying the RA (Andrich, 1988; Bond & Fox, 2007). Unlike classical test theory, which relies on sample-dependent statistics and ordinal raw scores, the RA provides interval-level measurement and estimates item difficulty and person ability on a common latent continuum. By ensuring that the scale operates as a coherent and invariant measure of ERF perception, RA strengthens its structural validity, interpretability, and generalizability across populations. Moreover, by meeting the requirements of invariance and additive structure, Rasch modeling aligns psychological measurement more closely with the standards of measurement used in the physical sciences.

### 1.5. The Present Study

In this study, children’s perceptions of ERF were explored by adapting an adult scale by Monsoon et al. (2022) and proposing the 10-item ERFQ-C. There were three aims. These aims are hierarchically organized: the primary goal of the study was the psychometric validation of the ERFQ-C, whereas the RA was intended to strengthen its measurement properties, and the path analysis (PA) was included as a secondary, exploratory step to examine theoretically relevant associations among constructs of ERF perception, malleability beliefs about emotions, and school-related well-being. We note that, although both ERF and malleability of emotions involve children’s subjective perceptions of emotion-related processes, the scales used to assess them target different aspects. Specifically, ERF was operationalized in terms of key components of flexibility (i.e., context sensitivity, repertoire, and feedback responsiveness), whereas malleability of emotions refers to beliefs about the changeability and controllability of emotions, thus targeting different levels of analysis.

1. The first aim was to examine the factorial structure of the ERFQ-C. We hypothesized that the scale would exhibit a unidimensional structure (Hypothesis 1a, H1a; Mignolli et al., 2025; Monsoon et al., 2022). Additionally, we expected the measure to demonstrate measurement invariance (MI) across sex (i.e., a binary classification given at birth; American Psychological Association, 2024) and school level (primary and lower secondary schools; H1b).
2. The second aim was to apply RA to the ERFQ-C after verifying its key assumptions (Andrich, 1988; Bond & Fox, 2007). We hypothesized that the assumptions of monotonicity, unidimensionality, and local independence would be satisfied (H2a). Furthermore, we expected the ERFQ-C to demonstrate adequate fit to the Rasch model (H2b). Finally, we expected the ERFQ-C to provide precise and invariant measurement, as evidenced by adequate person separation index (PSI) estimates, satisfactory person–item targeting, and the absence of significant differential item functioning (DIF; H2c).
3. The third aim was to investigate the mediating role of ERF perception in the relation between malleability beliefs of positive and negative emotions and school-related well-being. In line with the sparse literature on youth, we hypothesized that stronger malleability beliefs would be positively associated with higher perceived ERF (H3a; McKone et al., 2024), which in turn would be positively associated with greater school-related well-being (H3b; De France & Hollenstein, 2019; Haag et al., 2024; Parsafar et al., 2019; Quiñones-Camacho & Davis, 2018). Based on the literature on adults (Monsoon et al., 2022), we finally explored whether ERF perception would function as a partial or full mediator in this relation, while acknowledging limitations related to the cross-sectional design.

## 2. Materials and Methods

### 2.1. Participants and Procedure

The sample was comprised of 346 children attending primary school (fourth grade: *n* = 160, *M_age_* = 9.684, *SD* = 0.314, range: 8.967–10.665; 49.4% females) and lower secondary school (sixth grade: *n* = 105, *M_age_* = 11.665, *SD* = 0.385, range: 11.005–13.153; 51.4% females; seventh grade: *n* = 81, *M_age_* = 12.624, *SD* = 0.364, range: 12.052–14.005; 62.9% females) in Italy. In the whole sample, there were 184 males (53.2%) and 162 females (46.8%). Information on participants’ sex was provided by teachers before the data collection and was based on a binary classification (male/female) corresponding to sex assigned at birth (American Psychological Association, 2024). The study received approval from the Ethics Committee of the Department of Human Sciences of the University of Verona (protocol number 15396 dated 8 January 2025). After receiving authorization from the school head teachers, parents or legal guardians were asked to sign a written informed consent form for children’s participation. Data were collected using paper-and-pencil questionnaires administered during regular school classes. The research design was cross-sectional.

### 2.2. Measures

#### 2.2.1. Emotion Regulation Flexibility

ERF perception was assessed using the ERFQ-C, a child adaptation of the ERFQ developed by Monsoon et al. (2022) for adults (Table 1 reports the Italian version used for this study and its English translation). The ERFQ-C consists of 10 items (e.g., “I use many different strategies to regulate my emotions”) to be rated on a 5-point scale (1 = *not at all* and 5 = *very much*).

The measure was developed based on the Italian version of the ERFQ (IT-ERFQ; Mignolli et al., 2025), including, however, also the two items (numbers 1 and 8) of the ERFQ that had been eliminated in the IT-ERFQ. In addition, the original instructions were revised to include developmentally appropriate and easy-to-understand definitions of potentially complex concepts (i.e., “This questionnaire assesses how flexible you are in regulating and managing your emotions. Being flexible means having a variety of strategies available to choose from to regulate emotions, including knowing when to use the right strategy at the right time. Strategies are the different ways we use to regulate the intensity of emotions. The intensity of emotions is the strength with which we experience them: sometimes we feel strong emotions, other times we feel mild emotions. For each statement, select the number that best reflects how true the statement is for you.”).

The adaptation of the ERFQ for children was conducted by researchers with expertise in developmental and educational psychology, with the aim of ensuring age-appropriate wording and conceptual clarity. Although a formal pilot study or cognitive interviewing procedure was not conducted, the instrument was preliminarily reviewed with a small number of children in the upper age range of the target sample to verify general comprehensibility. During data collection, questionnaires were administered in classroom settings, with items read aloud by the researcher. Participants were explicitly invited to ask questions if they experienced difficulties in understanding any item or expression; however, no clarification requests were raised. Moreover, following the presentation of the revised instructions, participants were asked whether they had understood them, and they responded affirmatively.

Furthermore, given that the original instrument used a 7-point response scale, the number of response options was reduced to 5 to facilitate responding in younger participants. Cronbach’s (1951) alpha (α) was 0.887 and McDonald’s (1999) omega (ω) was 0.888, indicating a good internal consistency.

#### 2.2.2. Malleability of Emotions

Participants’ beliefs about the malleability of emotions were assessed through the Robust Incremental Beliefs about Emotion Scale for elementary school Children (RIBES-C; Raccanello et al., 2025). It comprises eight items pertaining to two factors, namely Malleability of positive emotions (four items; e.g., “I can learn to change the positive emotions I feel”) and Malleability of negative emotions (four items; e.g., “I can learn to change the negative emotions I feel”), to be rated on a 5-point scale (1 = *not at all* and 5 = *very much*). For the analyses, the scores were transformed into Rasch scores, following Raccanello et al. (2025). Internal consistency was satisfactory for each factor (α = 0.718/0.774; ω = 0.719/0.777).

#### 2.2.3. School-Related Well-Being

Participants’ well-being at school was measured using the School-related Well-Being Scale (SWBS; Stockinger et al., 2023; Italian adaptation by Raccanello et al., 2021). It is a single-factor measure with six items (e.g., “I feel good at school”) and a 5-point response scale (1 = *not at all* and 5 = *very much*). For the analyses, the scores were transformed into Rasch scores, following Raccanello et al. (2021). Internal consistency was good (α = 0.864; ω = 0.869).

### 2.3. Data Analysis

All the analyses were run using the R statistical environment (Version 4.6.0; R Core Team, 2026). Concerning the first aim, an exploratory structural equation modeling (ESEM) and a confirmatory factor analysis (CFA) were conducted using the *lavaan* package (Version 0.6.21; Rosseel, 2012). ESEM combines exploratory and confirmatory approaches within a unified framework, enabling us to avoid the reduction in statistical power that can result from splitting the sample (Asparouhov & Muthén, 2009). ESEM was implemented using the EFA-within-CFA framework available in *lavaan*. Preliminarily, the Kaiser–Meyer–Olkin (KMO) measure and the item-level measures of sampling adequacy (MSA) were utilized to assess the factorability of the correlation matrix (values ≥ 0.90 = excellent; 0.80–0.89 = very good; 0.70–0.79 = good; 0.60–0.69 = acceptable; 0.50–0.59 = poor; < 0.50 = unacceptable; Kaiser, 1974). Bartlett’s test of sphericity was used to examine whether the polychoric correlation matrix significantly differed from an identity matrix; a significant result indicates that the data are appropriate for factor extraction.

Given the ordered-categorical nature of the 5-point indicators, ESEM and CFA models were estimated treating items as ordered and using the diagonally weighted least squares (DWLS) estimator. Factors in the ESEM were rotated using oblique geomin rotation. The number of factors was determined using multiple retention criteria (package *parameters*, Version 0.29.1), and the final solution was selected based on convergence across methods, favoring parsimony in case of ties. Model fit was evaluated taking into account the comparative fit index (CFI), Tucker–Lewis index (TLI), root mean square error of approximation (RMSEA), and standardized root mean square residual (SRMR), following conventional guidelines (Hu & Bentler, 1999).

MI across sex and school level was tested via multi-group CFA (Putnick & Bornstein, 2016), sequentially examining configural, metric, and scalar invariance. Invariance was supported by changes in fit indices of ΔCFI ≤ 0.010, ΔRMSEA ≤ 0.015, and ΔSRMR ≤ 0.030 (metric) or ≤0.015 (scalar; Chen, 2007).

Regarding the second aim, the RA (Andrich, 1988; Bond & Fox, 2007) was applied using a partial credit model (PCM; Masters & Wright, 1997). Core assumptions were examined prior to model interpretation. Monotonicity was evaluated by inspecting the ordering of item thresholds. In Rasch models for polytomous items, thresholds are defined as the points on the latent trait continuum at which the probability of endorsing one response category equals that of endorsing the adjacent category. Thresholds partition the latent continuum into regions within which each response category is the most probable. Ordered thresholds indicate that response categories function as intended, reflecting increasing levels of the underlying trait. Homogeneity was assessed using the Martin-Löf (1966) test. Homogeneity in Rasch measurement refers to the property that all items included in a scale measure the same underlying latent trait and conform to a common measurement structure, such that item parameters are invariant across individuals differing in the level of the trait. Essential unidimensionality was further examined through principal component analysis (PCA) of Rasch-standardized residuals. Unidimensionality in RA refers to the assumption that responses to all items in a scale are governed by a single underlying latent trait, such that variation in item responses can be explained by differences in individuals’ locations on that single continuum. An eigenvalue of the first residual contrast below 2.000 was considered supportive of unidimensionality. Local independence was evaluated by inspecting residual correlations (Yen’s Q3-type). Local independence refers to the assumption that, conditional on the latent trait, responses to different items are statistically independent.

Model fit was assessed using Andersen’s (1973) likelihood-ratio test. Item and person fit were examined using infit mean square (infit-MNSQ) and outfit mean square (outfit-MNSQ), with acceptable values defined a priori between 0.700 and 1.300. Infit-MNSQ is a Rasch fit statistic that evaluates the degree to which observed responses conform to model expectations, with greater sensitivity to unexpected responses on items targeted to a person’s ability level. Outfit-MNSQ is a Rasch fit statistic that evaluates the degree of agreement between observed responses and model expectations, giving equal weight to all observations and thus being particularly sensitive to unexpected responses far from a person’s ability level.

Measurement precision and invariance were evaluated through the PSI (estimated from the PCM), targeting analysis, and DIF. PSI is a reliability statistic that quantifies a scale’s ability to discriminate between individuals with different levels of the latent trait. Targeting was examined using the person–item map and the distribution of person locations relative to item difficulty, as well as floor and ceiling effects. Targeting refers to the degree of alignment between the distribution of items and the distribution of persons on the same latent continuum. DIF for polytomous items was tested using ordinal logistic regression with total score as the matching variable, employing likelihood-ratio tests to examine sex and school level differences. DIF refers to the condition in which an item exhibits different measurement properties across distinct groups of respondents. *p*-values were adjusted using the Benjamini–Hochberg procedure. No purification was applied.

Raw scores were transformed into interval-level logit measures based on PCM person parameter estimates (Wright & Masters, 1982). Rasch-transformed scores were used in subsequent analyses for the ERFQ-C, RIBES-C, and SWBS scales, applying established conversion tables where appropriate (Raccanello et al., 2021, 2025).

Concerning the third aim, descriptive statistics and correlations among ERFQ-C, RIBES-C, and SWBS scores were computed, interpreting effect sizes according to Cohen (1988). A PA was then run to investigate the associations between RIBES-C and ERFQ-C, and, in turn, those between ERFQ-C and SWBS; moreover, the links between RIBES-C and SWBS were also calculated. Two multi-group PAs were conducted across sex and school level to examine the invariance of structural relations among the study variables. First, a configural model in which regression paths were freely estimated across groups was run. Subsequently, a constrained model was specified in which structural regression paths were held equal across groups, while intercepts and residual variances were allowed to vary. Model comparisons were performed using chi-square (χ^2^) difference tests under maximum likelihood estimation. When full structural invariance was not supported, partial invariance was examined using Wald tests to identify specific paths contributing to model misfit.

## 3. Results

### 3.1. Confirmatory Factor Analysis and Measurement Invariance (Aim 1)

Preliminary analyses indicated that the data were adequate for factor analyses: KMO was excellent (0.909), MSAs were very good (≥0.89), and Bartlett’s test was significant, χ^2^(45) = 1432.787, *p* < .001. Then, the number of factors to be retained in the ESEM was examined using 27 retention methods, i.e., optimal coordinates, acceleration factor, parallel analysis, Kaiser criterion, scree test based on standard error (*SE*), scree test based on *R*^2^, exploratory graph analysis using the graphical least absolute shrinkage and selection operator (EGA-glasso), exploratory graph analysis using the triangulated maximally filtered graph (EGA-TMFG), very simple structure with complexity 1 (VSS complexity 1), Velicer’s minimum average partial (MAP) test, and TLI. A one-factor solution was the most frequently supported (11 methods; 40.74% of all criteria), whereas the remaining methods suggested a range of alternative solutions, including two (three methods), three (two methods), four (four methods), five (two methods), six (three methods), and 10 factors (two methods). This pattern indicates a lack of convergence among multidimensional solutions, with no alternative dimensionality receiving comparable support. In line with the principle of parsimony and theoretical expectations regarding the unidimensional nature of ERF, the one-factor solution was retained. This decision was further supported by the good fit of the single-factor model in subsequent analyses and by convergent evidence from CFA and RA.

With a single-factor solution, ESEM and CFA are effectively equivalent because there are no cross-loadings to estimate and rotation is not defined. Therefore, parameter estimates and fit indices coincide (given the same estimator and identification). For both ESEM and CFA, the model showed good overall fit: CFI = 0.991, TLI = 0.988, RMSEA = 0.064, SRMR = 0.053. See Table 1 for factor loadings.

MI analyses across sex and school level are presented in Table 2. Changes in fit indices were small and within recommended thresholds, supporting both metric and scalar invariance across groups.

### 3.2. Rasch Analysis (Aim 2)

**Figure 1:**
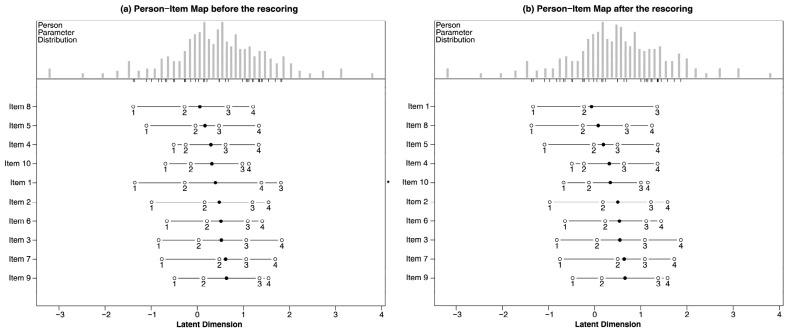
Person-item map for the 10 ERFQ-C items: (**a**) before rescoring; (**b**) after rescoring. The map displays persons’ ERF and items’ discriminatory capacity along the same latent dimension, with ERF ranging from lower (left) to higher (right) levels. Solid circles (•) indicate item locations, hollow circles (◦) thresholds, and asterisks (*) items with disordered thresholds.

First, regarding the core assumptions of the RA, inspection of item threshold estimates revealed that item 1 showed disordered thresholds (Figure 1a), suggesting a lack of monotonicity. Response frequencies for item 1 (1 = 31, 2 = 83, 3 = 147, 4 = 47, 5 = 38) indicated a relatively limited use of the highest category. However, disordered thresholds do not solely reflect low response frequencies; rather, they indicate that respondents do not use adjacent categories in a manner consistent with increasing levels of the latent trait. In the present case, the upper categories were not clearly differentiated, as respondents with higher levels of the construct did not consistently endorse the highest category over the adjacent one. This suggests that the distinction between the top response options is not psychometrically meaningful, thereby justifying the collapsing of these categories to restore proper threshold ordering. This is a standard procedure in Rasch modeling when adjacent categories are not functioning as intended. Therefore, item 1 was rescored from 1, 2, 3, 4, 5 to 1, 2, 3, 4, 4. After rescoring, a subsequent analysis confirmed monotonicity (Figure 1b): the thresholds became properly ordered, supporting the adequacy of the revised category structure. Importantly, this adjustment does not alter the substantive meaning of the item, but rather improves the measurement properties by ensuring that response categories reflect increasing levels of the latent trait. It is worth specifying that, with respect to interpretation and future use, the rescored item suggests that the distinction between the highest levels of endorsement is not psychometrically meaningful. Therefore, future applications of the ERFQ-C may consider either (a) using the revised 4-category structure for this item, or (b) retaining the original format while being aware that the top categories may function similarly, particularly in younger populations. This pattern may reflect limitations in the functioning of the response scale at the upper end of the continuum, rather than a limitation in the underlying construct.

The Martin–Löf test was non-significant, χ^2^(*df* = 38) = 32.354, *p* = .727, providing no evidence against unidimensionality. Moreover, the PCA of Rasch standardized residuals showed that the eigenvalue of the first residual contrast was 1.748 (subsequent contrasts: 1.447, 1.218, 1.155). Because it was below the commonly accepted threshold of 2.0, the results support the assumption of essential unidimensionality, indicating no substantive secondary dimension.

Finally, no item pairs showed residual correlations exceeding 0.30, suggesting adequate local independence. All residual correlations were below the commonly used threshold of 0.30, ranging from −0.274 to 0.136, indicating no evidence of substantial local dependence. The full residual correlation matrix is reported in Table 3. These results support the assumption of local independence for the ERFQ-C items.

Second, Andersen’s likelihood ratio test based on a random split was not significant, χ^2^(39) = 43.710, *p* = .278, supporting global model fit. In Table 4, the goodness-of-fit of each item examined using the infit-MNSQ and outfit-MNSQ statistics is reported. Overall, item fit was satisfactory. Infit-MNSQ ranged from 0.762 to 1.060 with a mean equal to 0.926, and outfit-MNSQ ranged from 0.768 to 1.058 with a mean equal to 0.943. No item showed a misfit according to the predefined criterion. Also, person fit, overall, was satisfactory. Infit-MNSQ ranged from 0.043 to 4.186, with a mean of 0.949, and outfit-MNSQ ranged from 0.043 to 5.030, with a mean of 0.943; 8.284% of the participants showed misfit according to the predefined criterion.

Third, concerning measurement precision and invariance, the results supported adequate model performance. The PSI was 0.848 (separation index = 2.362), indicating good discrimination among respondents. The distribution of person abilities largely overlapped the range of item difficulties, indicating adequate targeting. No floor effect was observed (0%), and the ceiling effect was minimal (0.870%), supporting adequate coverage at both ends of the scale. Importantly, the absence of floor effects suggests that the scale is sensitive to differences at the lower end of the latent trait, indicating that children with lower levels of ERF are adequately represented and differentiated in the present sample. Nevertheless, further research is needed to examine targeting in samples with a higher prevalence of low ERF levels (e.g., clinical populations), where more extreme item coverage may be required.

For sex and school level, respectively, no items showed evidence of DIF after Benjamini–Hochberg correction (all adjusted *p*s ≥ .370 for sex and ≥ .296 for school level). Effect sizes were negligible for all items (Nagelkerke’s *R*^2^ = 0.0002–0.0057 for sex; *R*^2^ = 0.0002–0.0058 for school level; all classified as “A” according to Jodoin and Gierl (2001), Zumbo (1999), and Zumbo and Thomas (1997) criteria), indicating MI across both sex and school level for this item set. Detailed DIF results are reported in Table 5.

Finally, to obtain Rasch-weighted scores, a raw total score-to-Rasch conversion table (Table 6) was constructed by grouping respondents according to their raw total score (the sum of item responses, excluding missing values). Then, for each raw total score, the mean Rasch score of the corresponding person location estimates was computed. Given that the instrument includes 10 items rated on a 5-point Likert scale, and that item 1 was rescored (i.e., response category 5 was recoded as 4), the total raw score ranges from 10 to 49. The table provides a transformation from raw total scores to Rasch person measures (logits). For practical reasons, the Rasch scores were linearly rescaled to the range 0–100 without altering their interval-level properties.

### 3.3. Path Analyses (Aim 3)

The correlations showed that all constructs were positively correlated with one another (see Table 7 for intercorrelations and descriptive statistics, calculated on the transformed Rasch scores for each of the three constructs). Correlations between ERFQ-C and malleability beliefs were large, as were correlations among the two malleability belief factors. The correlation between ERFQ-C and SWBS was moderate. The correlations between malleability belief factors and SWBS were small.

**Figure 2:**
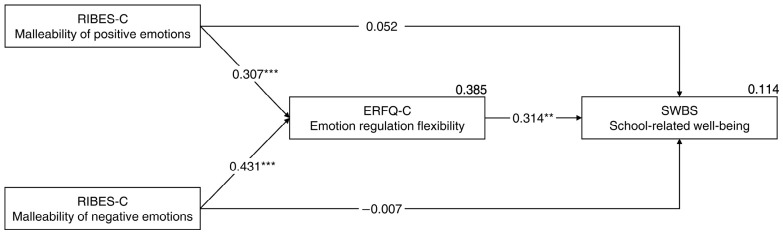
Path analysis between RIBES-C, ERFQ-C, and SWBS. ** *p* < .01. *** *p* < .001.

The path model was just-identified (*df* = 0) and therefore reproduced the observed covariance matrix exactly, yielding perfect fit indices (CFI = 1.00, TLI = 1.00, RMSEA = 0.00, SRMR = 0.00). As such, global fit indices are not informative in this case, and model evaluation relies on the interpretation of parameter estimates. The PA (Figure 2) indicated that beliefs about the malleability of both positive and negative emotions were positively associated with ERFQ-C scores. In turn, ERFQ-C was positively related to SWBS. The indirect effects of malleability beliefs about positive emotions on SWBS (β = 0.096, *SE* = 0.035, 95% CI [0.045, 0.182], *p* = .002) and of malleability beliefs about negative emotions on SWBS (β = 0.135, *SE* = 0.045, 95% CI [0.052, 0.230], *p* = .003) through ERFQ-C were significant. The direct effects of malleability beliefs on SWBS were not significant. These findings were consistent with a full mediation pattern of ERFQ-C in the relation between malleability beliefs and school-related well-being; however, given the cross-sectional design, these results do not imply causal mediation.

Finally, a multi-group PA across sex showed that constraining structural regression paths to equality did not significantly worsen model fit relative to the configural model, Δχ^2^(5) = 8.793, *p* = .118, suggesting structural invariance across sex. Across school levels, the multi-group PA, constraining structural paths, resulted in a significant decrease in fit, Δχ^2^(5) = 12.905, *p* = .024, indicating that at least one path differed between groups. Wald tests revealed that the path from malleability of positive emotions to ERFQ-C significantly varied across groups, χ^2^(1) = 10.351, *p* = .001, whereas the remaining paths did not (*p*s ≥ .168). A partially invariant model, allowing this path to vary while constraining the others, did not significantly differ from the configural model, Δχ^2^(4) = 3.939, *p* = .414, supporting partial structural invariance. The association between malleability of positive emotions to ERFQ-C was stronger in younger students (β = 0.415, *p* < .001) than in older students (β = 0.226, *p* = .012).

## 4. Discussion

The emerging focus of research on youth’s ERF (Aldao et al., 2015; Bonanno & Burton, 2013; Braet et al., 2026; Haag et al., 2024) permits advancing both theoretical and applied knowledge to inform actions that accompany children on their developmental journey through their emotions and support their well-being. In this work, an adaptation of the ERFQ (Mignolli et al., 2025; Monsoon et al., 2022) for children, the ERFQ-C, was developed to offer a brief, age-appropriate, and valid instrument respecting the properties of fundamental measurement (Andrich, 1988; Bond & Fox, 2007), to be used in contexts in which there is still a lack of such instruments.

### 4.1. Theoretical and Practical Implications

The process of adapting the ERFQ for children included changes aimed at making the instrument accessible to younger participants, starting from the already Italian-validated version (IT-ERFQ; Mignolli et al., 2025). Following basic criteria commonly used in developmental psychology, item wording was simplified, explicit definitions of ER strategies and ERF were added in the instructions, and the response options were reduced from 7 to 5. Then, its factorial properties were tested (first aim), corroborating the unidimensional structure already observed in the original and the Italian adult versions (Mignolli et al., 2025; Monsoon et al., 2022) and confirming H1a. The ERFQ-C also demonstrated MI across sex and school level, supporting the stability of its unidimensional structure and indicating that it assesses perceived ERF equivalently across these groups (confirming H1b). This provides evidence of score comparability and supports its use in future group-level comparisons (Putnick & Bornstein, 2016).

When analyzed with Rasch modeling (Andrich, 1988; Bond & Fox, 2007; second aim), the ERFQ-C showed strong psychometric properties. The analyses indicated that the scale satisfied the assumptions of monotonicity, unidimensionality, and local independence (confirming H2a). After revealing an appropriate fit to the Rasch model (confirming H2b), the data supported the view that ERFQ-C functions as a precise and invariant measure, with adequate PSI, good targeting, and no evidence of DIF by sex or school level (confirming H2c). By providing interval-level scores on a common latent continuum, this approach enhances the scale’s interpretability and supports its generalizability across populations. This means that comparisons between individuals do not depend on the particular items administered, and item calibrations do not depend on the specific sample. This invariance supports the additivity of measures and justifies the use of meaningful quantitative operations. Consequently, RA enables ERF perception to be assessed in a manner that approximates the standards of measurement in the physical sciences, thereby enhancing the validity, reliability, and interpretability of the resulting scale.

Examining the associations between perceived ERF and the other two health-related constructs, i.e., malleability beliefs about emotions and school-related well-being, through a PA, prior literature was extended and overall support of previous results about both adults and children/adolescents was found (third aim). Importantly, the ERFQ-C captures children’s self-reported representations of their regulatory flexibility. In middle childhood, such representations may play a particularly central role, as regulatory processes are still developing and are often mediated by children’s perceptions of their own abilities, effort, and effectiveness (Garnefski et al., 2001; Harley et al., 2019; Pekrun, 2006, 2024; Phillips & Power, 2007; Zimmer-Gembeck & Skinner, 2011). In this sense, the present findings should be interpreted as reflecting perceived ERF, which may influence emotional and behavioral outcomes even when actual regulatory flexibility is not yet fully consolidated.

On the one hand, children who perceived both positive and negative emotions as more malleable were more likely to exhibit a perceived higher ability in ERF (confirming H3a). This was in line with Monsoon et al.’s (2022) findings about the positive link between adults’ emotion control and ERF perception, and McKone et al.’s (2024) findings, which showed that adolescent girls perceiving a stronger control on stressors prefer some strategies (e.g., problem-solving, seeking support) rather than others (e.g., reframing, acceptance). On the other hand, children with a stronger perception of their ability in ERF tended to have higher scores in school-related well-being (confirming H3b). While, to our knowledge, no previous research examined this association focusing on this type of well-being, this confirms results from studies also with youth, which revealed positive associations between ERF perception and various indicators of well-being (e.g., De France & Hollenstein, 2019; Haag et al., 2024; Parsafar et al., 2019; Quiñones-Camacho & Davis, 2018). Finally, the possible mediating role of ERF in the relation between malleability beliefs and school-related well-being was considered, suggesting a pattern consistent with full mediation, although causal interpretations cannot be drawn due to the cross-sectional design. This is coherent with previous findings suggesting a mediating role of ERF perception in adult samples in the relation between emotion control and distress (Monsoon et al., 2022). Moreover, while relations between malleability beliefs and both ER strategies and well-being had already been demonstrated with youth (Crawford et al., 2021; De France & Hollenstein, 2021; Dickerson et al., 2018; Ford et al., 2018; Jiang et al., 2022; McLachlan et al., 2022; Raccanello et al., 2025; Romero et al., 2014; Schleider & Weisz, 2016; Skymba et al., 2022), to our knowledge, no attention had been previously paid to its associations with perceived ERF. However, the data also indicated no direct significant effect of malleability beliefs on school-related well-being, highlighting the potential role of ERF perception in accounting for the association between these beliefs and students’ well-being, although alternative explanations cannot be ruled out given the cross-sectional design and the use of self-report measures. While these relations were invariant across males and females, a weaker link between the malleability of positive emotions and perceived ERF was observed among older students. This could be because, for older students, negative emotions become more salient (Maciejewski et al., 2017), and therefore the link between the malleability of positive emotions and ERF may be less relevant. Nevertheless, further research is needed to confirm this trend.

At the applied level, the results of this research suggest that higher levels of self-reported ERF are associated with more favorable indicators of school-related well-being. However, the complexity of distinguishing between ER variability, flexibility, and adaptation in relation to an individual’s goals is acknowledged (Aldao et al., 2015). Therefore, even if the findings of this study may tentatively indicate that professionals could consider sustaining both students’ malleability beliefs and ERF as potentially relevant targets to support school-related well-being, the fact that ERF is adaptive per se cannot be assumed, independent of the fit between one’s own characteristics, goals, and contextual demands.

Recognizing this, the present results may inform future research on interventions aimed at improving children’s ERF, focusing on a variety of components spanning from context sensitivity, repertoire, and feedback responsiveness (Bonanno & Burton, 2013; Kalokerinos & Koval, 2024). For example, children might, in principle, benefit from being supported to know and use context-sensitive combinations of ER strategies (Braet et al., 2026). Moreover, the possibility to modify malleability beliefs about emotions to make them more incremental (McLachlan et al., 2022; Smith et al., 2018; Specker & Nickerson, 2024) represents a theoretically grounded avenue that may be relevant for ERF and, in turn, school-related well-being, but that warrants further empirical testing. At the same time, improvements in ERF as measured by the ERFQ-C could be expected, based on theory, to relate to emotional intelligence (Mayer et al., 2016), increasing the likelihood that individuals apply ER strategies in response to both contextual and situational cues and to their goals (Aldao et al., 2015). More broadly, it should be noted that the ERFQ-C captures a self-reported operationalization of ERF, which may not fully reflect the underlying dynamic regulatory processes.

From this perspective, the ERFQ-C can be understood as capturing ERF at the level of children’s subjective representations. Rather than directly indexing regulatory behavior, the instrument provides insight into how children conceptualize and evaluate their own regulatory capacities. This level of analysis is theoretically meaningful, as such representations are known to guide engagement in regulation, strategy selection, and emotional responses, particularly in developmental stages in which regulatory skills are still emerging. In this sense, a key contribution of the present study lies in advancing how complex regulatory constructs such as ERF can be operationalized in childhood, providing a basis for future longitudinal, experimental, and clinical research.

### 4.2. Limitations and Future Directions

The study suffers from some limitations. First, ERF perception was operationalized using a self-report instrument, which is prone to memory and compliance biases. Although self-reports provide access to individuals’ subjective perceptions that are difficult to capture from the outside (Pekrun, 2020), future studies should triangulate the research design by implementing other assessment methods, such as the computational tools suggested by Aldao et al. (2015) and already implemented using EMA with adolescents (e.g., Braet et al., 2026). Moreover, although malleability beliefs and perceived ERF are conceptually distinct, both were assessed through self-report measures administered in the same session. Therefore, shared method variance and some conceptual proximity between the constructs may have contributed to the observed associations and remain important explanatory factors, warranting a cautious interpretation of the PA findings. The issue about shared variance also regards the measurement of well-being. Future research should adopt multi-method approaches to better disentangle these constructs. Second, although the ERFQ-C was adapted to ensure age-appropriate wording and supported during administration through read-aloud procedures and opportunities for clarification, no formal qualitative piloting or cognitive interviewing was conducted to systematically assess item comprehension. Therefore, differences in developmental comprehension cannot be ruled out, particularly for items requiring more advanced reflection on regulatory processes, such as monitoring the duration of strategy use or coordinating multiple strategies across situations. Accordingly, it cannot be entirely excluded that some items may have been interpreted differently across participants, especially among younger ones. Future research should incorporate qualitative methods to further examine how children understand and respond to the instrument. Moreover, while the ERFQ instructions were integrated, adding explicit definitions of ER and ERF to the ERFQ-C, future applications could include specific examples of ER strategies to help, especially younger children, better contextualize what is being measured. Third, a cross-sectional design was used, which limits causal inference. Future research should assess malleability beliefs, ERF perception, and well-being at multiple time points. Fourth, malleability beliefs, ERF perception, and school-related well-being were measured at different levels of generality. In the future, maintaining the same level of granularity across all variables could help identify stronger relations. Fifth, non-clinical participants were considered. Future investigations could include children from clinical populations to gather data on mental health, which could serve as the basis for interventions. Sixth, only children attending school in Italy, a Western, educated, industrialized, rich, and democratic (WEIRD) country, were recruited. Future research should examine the psychometric properties of the ERFQ-C in more diverse samples, including underrepresented populations.

## 5. Conclusions

The concept of ERF has enabled a broader understanding of the impact of ER in everyday life by systematically emphasizing that no unique ER strategy is inherently adaptive; rather, its effectiveness depends on its fit with individual characteristics and contextual demands. To advance ER research from a developmental perspective, a measure for assessing perceived ERF in children, the ERFQ-C, was introduced, leveraging the advantages of Rasch modeling. Moreover, the observed pattern of associations involving children’s ERF perception suggests its potential relevance for their well-being and highlights possible pathways through which it may be enhanced.

## Figures and Tables

**Table 1:** Item description in the English and Italian versions and factor loadings (ESEM/CFA) for the ERFQ-C scale.

Item Number	English Version	Italian Version	Loadings
1	I have many strategies I can choose from to manage my emotions.	Ho molte strategie tra cui posso scegliere per gestire le mie emozioni.	0.770
2	When I want to manage an emotion, I know exactly what strategy(s) to use.	Quando voglio gestire un’emozione, so esattamente che strategia/e usare.	0.722
3	I use many different strategies to regulate my emotions.	Uso tante strategie diverse per regolare le mie emozioni.	0.772
4	When an emotion regulation strategy does not work, I try to use a different one.	Quando una strategia di regolazione delle emozioni non funziona, provo a usarne una diversa.	0.633
5	I use different types of emotion regulation strategies for different types of situations or problems.	Uso diversi tipi di strategie di regolazione delle emozioni per diversi tipi di situazioni o problemi.	0.645
6	I combine different types of regulation strategies when trying to manage my emotions (for example, using different strategies together or using them one after the other).	Combino diversi tipi di strategie di regolazione quando provo a gestire le mie emozioni (per esempio, usando diverse strategie insieme oppure usandole una dopo l’altra).	0.590
7	If one emotion regulation strategy doesn’t work, I usually know what to use instead.	Se una strategia di regolazione delle emozioni non funziona, di solito so cosa usare al suo posto.	0.653
8	The emotion regulation strategies that I use usually work well for me.	Le strategie di regolazione delle emozioni che uso di solito funzionano bene per me.	0.667
9	When I use an emotion regulation strategy, I know for how long it is better to use it.	Quando uso una strategia di regolazione delle emozioni, so per quanto tempo è meglio usarla.	0.580
10	I use different emotion regulation strategies depending on the type or intensity of the emotion.	Uso diverse strategie di regolazione delle emozioni a seconda del tipo o dell’intensità dell’emozione.	0.651

**Table 2:** Results of measurement invariance analyses across sex and school level.

Groups	Model Comparison	ΔCFI	ΔRMSEA	ΔSRMR
Sex	Configural → Metric	−0.005	0.012	0.009
	Metric → Scalar	0.005	−0.023	−0.008
School level	Configural → Metric	−0.005	0.011	0.009
	Metric → Scalar	0.006	−0.023	−0.008

*Note. N* = 346. ΔCFI/RMSEA/SRMR = change in comparative fit index/root-mean-square error of approximation/standardized root mean square residual.

**Table 3:** Residual correlation matrix (ERFQ-C items).

Item Number	1	2	3	4	5	6	7	8	9	10
1	—	0.134	0.136	−0.099	−0.152	−0.234	−0.095	−0.002	−0.209	−0.154
2		—	0.005	−0.226	−0.239	−0.165	−0.030	0.000	−0.085	−0.274
3			—	−0.196	−0.050	−0.174	−0.173	−0.018	−0.132	−0.183
4				—	−0.089	−0.043	−0.085	−0.201	−0.166	0.022
5					—	−0.125	−0.193	0.026	−0.238	0.020
6						—	−0.101	−0.198	0.050	−0.157
7							—	−0.120	−0.122	−0.122
8								—	−0.215	−0.211
9									—	−0.023
10										—

*Note.* Values represent residual correlations (Yen’s Q3) from the partial credit model. Only the upper triangular matrix is reported. All residual correlations were below the commonly used threshold of 0.30 (range: −0.274 to 0.136), indicating no evidence of substantial local dependence and supporting the assumption of local independence for the ERFQ-C items.

**Table 4:** Infit-MNSQ and outfit-MNSQ of each item of the ERFQ-C scale.

Item Number	Infit-MNSQ	Outfit-MNSQ
1	0.775	0.784
2	0.870	0.883
3	0.762	0.768
4	0.973	1.029
5	0.959	1.000
6	1.040	1.035
7	0.919	1.037
8	0.944	0.937
9	1.060	1.058
10	0.957	0.902

**Table 5:** Differential item functioning (DIF) results for ERFQ-C items across sex and school level.

Item Number	χ^2^ (LRT)	*p*	*p* (adj)	*R* ^2^	Effect Size
1	0.49/2.71	.780/.258	.842/.629	0.0003/0.0015	Negligible/Negligible
2	2.29/0.39	.318/.822	.636/.822	0.0010/0.0002	Negligible/Negligible
3	0.40/1.95	.817/.378	.842/.629	0.0002/0.0009	Negligible/Negligible
4	4.04/5.66	.132/.059	.384/.296	0.0018/0.0037	Negligible/Negligible
5	6.59/2.00	.037/.367	.370/.629	0.0057/0.0008	Negligible/Negligible
6	0.41/1.37	.816/.505	.842/.647	0.0002/0.0008	Negligible/Negligible
7	0.34/0.64	.842/.725	.842/.805	0.0003/0.0004	Negligible/Negligible
8	4.42/2.80	.109/.247	.384/.629	0.0030/0.0017	Negligible/Negligible
9	3.74/1.32	.154/.518	.384/.647	0.0030/0.0008	Negligible/Negligible
10	1.51/6.15	.471/.046	.785/.296	0.0014/0.0058	Negligible/Negligible

*Note.* χ^2^(LRT) = likelihood-ratio test statistic from ordinal logistic regression; *p* (adj) = *p*-values adjusted using the Benjamini–Hochberg procedure; *R*^2^ = Nagelkerke’s *R*^2^ as an effect size index. Values are reported as sex/school level. Effect sizes were classified according to established criteria (Jodoin & Gierl, 2001; Zumbo, 1999). All values indicate negligible DIF.

**Table 6:** Conversion table from raw total scores of the items of the ERFQ-C scale to logit scores, then rescaled from 0 to 100.

Raw Total Scores	Logit Rasch Scores	Logit Rasch Scores (0–100)
10	−3.974	0.000
11	−3.184	9.258
12	−2.464	17.701
13	−2.030	22.776
14	−1.715	26.477
15	−1.463	29.427
16	−1.251	31.906
17	−1.067	34.063
18	−0.903	35.987
19	−0.754	37.737
20	−0.616	39.352
21	−0.487	40.861
22	−0.365	42.286
23	−0.249	43.643
24	−0.138	44.945
25	−0.031	46.203
26	0.072	47.427
27	0.175	48.623
28	0.275	49.798
29	0.374	50.959
30	0.472	52.111
31	0.570	53.260
32	0.669	54.412
33	0.768	55.573
34	0.868	56.749
35	0.970	57.947
36	1.075	59.177
37	1.184	60.447
38	1.297	61.771
39	1.416	63.164
40	1.542	64.646
41	1.679	66.244
42	1.828	67.996
43	1.995	69.958
44	2.188	72.215
45	2.419	74.914
46	2.710	78.328
47	3.115	83.075
48	3.803	91.135
49	4.560	100.000

**Table 7:** Intercorrelations and descriptive statistics (ranges; means, *M*; standard deviations, *SD*; 95% confidence intervals, CI) for the ERFQ-C, the two dimensions of the RIBES-C, and the SWBS.

	1	2	3	4
1. ERFQ-C	-			
2. RIBES-C—Malleability of positive emotions	0.538 ***	-		
3. RIBES-C—Malleability of negative emotions	0.512 ***	0.642 ***	-	
4. SWBS	0.337 ***	0.207 ***	0.201 ***	-
Range	0–100	1–10	1–10	1–10
*M*	51.582	6.009	5.617	5.706
*SD*	13.015	1.561	1.748	1.731
95% CI	[50.172, 52.991]	[5.839, 6.177]	[5.427, 5.806]	[5.518, 5.893]

*Note.* *** *p* < .001. ERFQ-C scores were transformed to Rasch logits and linearly rescaled to 0–100, whereas RIBES-C and SWBS scores were Rasch-transformed using their established published conversion tables and retained on a 1–10 metric.

## Data Availability

The original data presented in the study are openly available in Open Science Framework at https://osf.io/b3579/overview?view_only=9b840f52d92a438d927c3138b58e32e5 (accessed on 26 May 2026).
